# The prevalence of bifid iliopsoas tendon on MRI in children

**DOI:** 10.1007/s11832-014-0596-x

**Published:** 2014-06-01

**Authors:** Thomas Crompton, Claire Lloyd, Michail Kokkinakis, Fabian Norman-Taylor

**Affiliations:** 1 Department of Paediatric Orthopaedic Surgery, Evelina London Children’s Hospital Guy’s and St Thomas’ NHS Foundation Trust, London, UK; 2 Department of Paediatric Radiology, Evelina London Children’s Hospital Guy’s and St Thomas’ NHS Foundation Trust, London, UK; 3 14b Belmont Hill, London, SE13 5BD UK

**Keywords:** Iliopsoas, Tendon, Hip, MRI, Children, Paediatric orthopaedics

## Abstract

**Objective:**

The variation in the anatomy of the iliopsoas tendon is important information for orthopaedic surgeons operating around the hip. The aim of this study was to identify the prevalence of bifid iliopsoas tendons in children on magnetic resonance imaging (MRI).

**Methods:**

MRI hip and pelvis images of 50 sequential children aged 7–15 years were retrieved from our radiology database at the Evelina London Children’s Hospital from 2007 to 2013. Included were 37 children with imaging of both hips and 13 children with imaging of one hip only. Therefore, our study was based on a total of 87 hips.

**Results:**

At least 1 bifid tendon was noted in 13 children (26 %). Five children from a total of 37 (14 %) with both hips adequately imaged had bilateral bifid tendons. Among all 87 adequately imaged hips, 18 (21 %) were found to have two discrete distal iliopsoas tendons.

**Conclusions:**

Bifid iliopsoas tendon is noted anecdotally by surgeons but was only reported in scattered case reports and a few anatomical studies until very recently. Our finding is that a bifid iliopsoas tendon with two distinct tendinous components at the level of the hip joint is quite common. This has clinical significance, particularly in children’s orthopaedic surgery when an adequate iliopsoas release is important.

## Background

The iliopsoas tendon is the conjoint tendon of the psoas major and iliacus muscles. The psoas major—a long, fusiform muscle—originates from the transverse processes of the lumbar vertebrae and runs caudally across the brim of the pelvis to lie anterior to the hip capsule before inserting into the lesser trochanter of the femur. The iliacus is a flat, triangular muscle arising from the upper two-thirds of the iliac fossa, the inner lip of the iliac crest and the front of the hip capsule. The fibres converge with the tendon of the psoas major. A bursa separates the tendon from the pubis and the hip capsule [1].

In children with cerebral palsy and other neuromuscular conditions, contractures of the iliopsoas, and other muscles, are common. This is due to disuse, muscular imbalance or spasticity. A distal iliopsoas release is performed as part of many hip procedures in these children [2]. Iliopsoas contractures cause anterior pelvic tilt and a lack of hip extension, contributing to a crouch gait. Contractures of iliopsoas, if severe and persistent, can lead to hip displacement. Iliopsoas tenotomy can be combined with adductor release as part of a medial release for preventing spastic hip displacement [3–7]. It can be combined with sartorius and/or rectus femoris release as part of an anterior release for hip flexion contracture. Iliopsoas tenotomies are also performed in open or closed reduction for developmental dysplasia of the hip (DDH) [8, 9].

The iliopsoas tendon has been implicated as a generator of hip pain and a cause of labral injury due to impingement [10]. This pathology is defined as coxa saltans or snapping hip syndrome. The two bony structures commonly implicated are the anterior aspect of the femoral head and the joint capsule and the iliopectineal eminence of the pelvic brim [11–13]. It has also been described as a source of pain following total joint replacement [14]. Open and arthroscopic release of distal iliopsoas tendons are frequently performed [15–17].

It is our own intraoperative observation that a bifid iliopsoas tendon is frequently present. This surgical observation has only been supported by scattered case reports in the international literature until recently [10, 18–20]. A fatty cleft between the iliopsoas tendon and a distinct tendon within the lateral part of the iliacus muscle has previously been described on MR hip arthrograms in adults [21].

Our hypothesis was that a bifid tendon is more common than previously reported, so the aim of this study was to identify the prevalence of bifid iliopsoas tendons visible on MRI in children.

## Methods

We carried out a retrospective study on paediatric hip MRI scans performed at the Evelina London Children’s Hospital from 2007 to 2013. Only children with adequate imaging of at least one iliopsoas tendon were included in the study. Patients in hip spica casts or with extreme movement artefacts were excluded as the tendon could not be adequately visualised. Patients with previous hip surgery were also excluded.

The MRI protocol included coronal STIR, coronal T1- and transverse T1-weighted sequences in all cases. In some cases a transverse gradient-echo T2-weighted sequence was also performed. The scans were reviewed for the presence of a bifid iliopsoas tendon by a paediatric consultant radiologist (CL). All of the studies were performed on either a 1.5-T Siemens Avanto or a 1.5-T Philips Achieva scanner using a body coil.

**Fig. 1:**
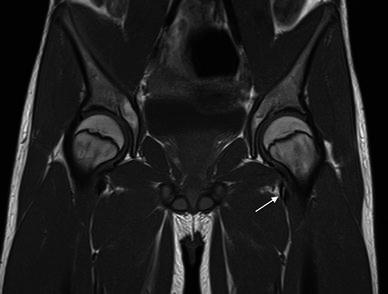
Coronal T1-weighted image, with *arrow* showing the bifid iliopsoas tendon at level of the femoral neck

**Fig. 2:**
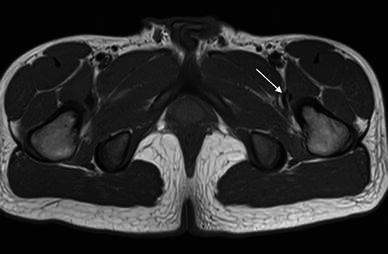
Transverse T1-weighted image in the same patient, showing the bifid iliopsoas tendon

**Fig. 3:**
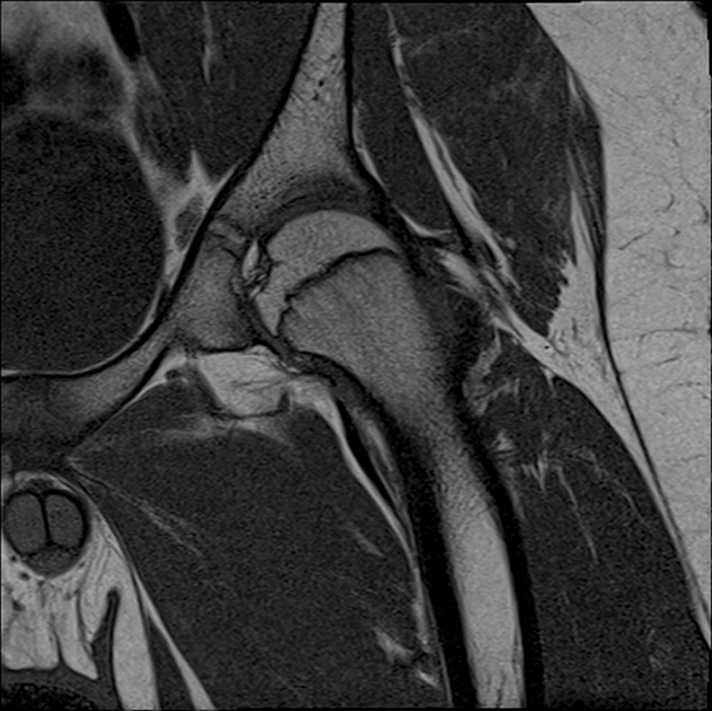
Coronal T1-weighted image of the left hip, showing a single iliopsoas tendon (no bright, fatty cleft separating it in two)

We identified 50 children aged 7–15 years (mean 11.9) who fulfilled the above inclusion criteria. Thirteen patients had only one hip imaged. Thirty-seven patients had both hips imaged. Consequently, 87 hips were assessed in the study. Bifid tendons were reported when two separate low-signal intensity tendons separated by a fatty cleft were noted at the level between the femoral neck and the lesser trochanter. Examples of transverse and coronal T1-weighted images are shown in Figs. 1 and 2 from a patient in our series. Figure 3 is an image of the left hip showing a single iliopsoas tendon in a patient from our series.

## Results

Among all of the 87 adequately imaged hips there were 18 (21 %) with two discrete distal iliopsoas tendons. At least one bifid tendon was noted in 13 children (26 % of children). Five children (14 %) were shown to have a bilateral bifid iliopsoas tendon.

## Discussion

The iliopsoas tendon is regarded as the conjoint tendon of the psoas major and iliacus [1]. Two anatomical studies were previously performed to analyse the cross-section of the iliopsoas musculotendinous unit at the site of arthroscopic tenotomies, but no bifid iliopsoas tendons were reported [22, 23]. In another anatomic dissection of 24 embalmed hips, Tatu et al. showed that three separate elements combine to form the iliopsoas musculotendinous unit [17]: the main psoas major tendon, an accessory tendon arising from the iliacus muscle, and muscular fibres belonging to the iliacus muscle that attach to the lesser trochanter without tendinous processes. The accessory iliacus tendon fuses progressively with the main psoas tendon. An absence of fusion was not detected in any of the specimens. Interestingly, in two cases, the main psoas major tendon was completely split into two bundles, and a partial split was observed in a further two cases. Guilin et al. [20] reported one hip with a bifid psoas major in a dynamic sonographic study of 42 hips of healthy volunteers that was performed in an attempt to observe the components Tatu described. A very recent anatomical study in which 53 fresh frozen adult cadavers were examined specifically for variation in the anatomy of the iliopsoas tendon showed that the majority of the iliopsoas tendons (64 %) were bifid at the level of the hip joint [10]. They also showed that 7.5 % of their cadavers had three distal iliopsoas tendons. These anatomical studies in adults support our findings of a bifid tendon. Based on these limited reports, a bifid iliopsoas tendon probably arises as a constitutional anatomical variant. Polster et al. [21] found a longitudinal cleft of increased T1 signal in the psoas tendon in 14 of 20 adult patients undergoing a hip MR arthrogram.

Bifid iliopsoas as the aetiology of snapping hip syndrome, coxa saltans [11–13], was reported by Deslandes et al. after using ultrasound to dynamically evaluate 18 patients who presented clinically with unilateral or bilateral snapping hips [22]. Two patients with a total of three snapping hips had a bifid psoas tendon bilaterally. The cause of the snapping hip was flipping of the bifid heads over one another.

Shu and Safran [18] reported a case of an 18-year-old softball player who underwent arthroscopic distal release of the iliopsoas tendon but continued to have painful snapping postoperatively. A revision arthroscopic procedure revealed a bifid iliopsoas tendon. He remained asymptomatic following division of both tendon heads. They concluded that the differential diagnosis of failed iliopsoas lengthening surgery should include the consideration of incomplete release due to the presence of a bifid iliopsoas tendon. Consequently, this anatomical variation is of clinical significance for both adult and children’s orthopaedics.

Paediatric hip surgeons must take this into account in both neuromuscular and developmental hip dysplasia when they perform iliopsoas tenotomies. Knowledge of the possibility of two discrete iliopsoas tendons can prevent incomplete release. Adequate iliopsoas exposure and identification of a bifid tendon should be performed in all cases. When a distal iliopsoas tenotomy is carried out but the release seems inadequate on examination, this should alert the surgeon to the possibility of a second intact band of iliopsoas. Also, if the tendon appears too small, this should prompt the surgeon to seek another tendon. In adult orthopaedics, surgeons might face difficulties with arthroscopic iliopsoas tenotomies when two distal tendons are present. Preoperative MRI scans should be reviewed in this regard to be prepared.

Philippon et al. reported a prevalence of two discrete tendons of 64 % in their cadaveric study. Our study demonstrated that, on MRI scanning, 13 of 50 children (26 %) had at least one bifid iliopsoas tendon. It may be that MRI is not sensitive enough to detect all of the bifid tendons, and that a bifid tendon is even more common than this in children. Both studies confirm a higher prevalence of bifid iliopsoas tendons than stated in earlier reports in the literature.

Limitations of our study include that it includes a relatively small number of patients. Although we demonstrated the presence of a bifid tendon at the level of the hip joint, we still do not know whether this is a split of the psoas major tendon or a lack of fusion of the psoas and iliacus tendons. To correctly identify this we would need pelvic and lumbar MRI scans on all investigated patients. This is of anatomic relevance but probably not of surgical or clinical importance. In addition, the children involved underwent an MRI scan for presumed pelvic or hip pathology, and although it is hard to link a bifid tendon to any of the reported symptoms, this cannot be considered an entirely healthy paediatric population.

## Conclusion

Bifid iliopsoas tendon was only reported in scattered case reports until very recently [10, 18, 21, 24]. The findings of this study confirm that the prevalence of a bifid iliopsoas tendon with two distinct tendinous components at the surgically important level between the femoral neck and the lesser trochanter is quite high. Knowledge of the prevalence of normal variation of the iliopsoas tendon is useful to orthopaedic surgeons operating around the hip in young adults, adolescents and children. This anatomical variant is visible on standard MRI scanning.
